# Decreased expression of long noncoding RNA GAS5 indicates a poor prognosis and promotes cell proliferation in gastric cancer

**DOI:** 10.1186/1471-2407-14-319

**Published:** 2014-05-06

**Authors:** Ming Sun, Fei-yan Jin, Rui Xia, Rong Kong, Jin-hai Li, Tong-peng Xu, Yan-wen Liu, Er-bao Zhang, Xiang-hua Liu, Wei De

**Affiliations:** 1 Department of Biochemistry and Molecular Biology, Nanjing Medical University, Nanjing 210029, People’s Republic of China; 2 Department of General Surgery, First Affiliated Hospital, Nanjing Medical University, Nanjing, People’s Republic of China; 3 Department of Oncology, First Affiliated Hospital, Nanjing Medical University, Nanjing, People’s Republic of China

**Keywords:** Gastric cancer, Long noncoding RNA, GAS5, Poor prognosis, Cell proliferation

## Abstract

**Background:**

Gastric cancer is the second leading cause of cancer death and remains a major clinical challenge due to poor prognosis and limited treatment options. Long noncoding RNAs (lncRNAs) have emerged recently as major players in tumor biology and may be used for cancer diagnosis, prognosis, and potential therapeutic targets. Although downregulation of lncRNA GAS5 (Growth Arrest-Specific Transcript) in several cancers has been studied, its role in gastric cancer remains unknown. Our studies were designed to investigate the expression, biological role and clinical significance of GAS5 in gastric cancer.

**Methods:**

Expression of GAS5 was analyzed in 89 gastric cancer tissues and five gastric cancer cell lines by quantitative reverse-transcription polymerase chain reaction (qRT-PCR). Over-expression and RNA interference (RNAi) approaches were used to investigate the biological functions of GAS5. The effect of GAS5 on proliferation was evaluated by MTT and colony formation assays, and cell apoptosis was evaluated by hochest stainning. Gastric cancer cells transfected with pCDNA3.1 -GAS5 were injected into nude mice to study the effect of GAS5 on tumorigenesis *in vivo*. Protein levels of GAS5 targets were determined by western blot analysis. Differences between groups were tested for significance using Student’s *t*-test (two-tailed).

**Results:**

We found that GAS5 expression was markedly downregulated in gastric cancer tissues, and associated with larger tumor size and advanced pathologic stage. Patients with low GAS5 expression level had poorer disease-free survival (DFS; P = 0.001) and overall survival (OS; P < 0.001) than those with high GAS5 expression. Further multivariable Cox regression analysis suggested that decreased GAS5 was an independent prognostic indicator for this disease (P = 0.006, HR = 0.412; 95%CI = 2.218–0.766). Moreover, ectopic expression of GAS5 was demonstrated to decrease gastric cancer cell proliferation and induce apoptosis *in vitro* and *in vivo*, while downregulation of endogenous GAS5 could promote cell proliferation. Finally, we found that GAS5 could influence gastric cancer cells proliferation, partly via regulating E2F1 and P21 expression.

**Conclusion:**

Our study presents that GAS5 is significantly downregulated in gastric cancer tissues and may represent a new marker of poor prognosis and a potential therapeutic target for gastric cancer intervention.

## Background

Gastric cancer is the second leading cause of cancer death, and is the most common gastrointestinal malignancy in East Asia, Eastern Europe, and parts of Central and South America [1]. Although the majority of the patients at an early stage of gastric carcinoma can be cured by surgery, more than half of those at an advanced stage of the disease die of carcinoma recurrence, even after undergoing curative gastrectomy [2]. Therefore, better understanding of the pathogenesis and identification of the molecular alterations is essential for the development of useful indicators that aid novel effective therapies for gastric cancer [3-5].

It is well known that protein-coding genes account for only 2% of the total genome, whereas the vast majority of the human genome can be transcripted into noncoding RNAs [6-9]. Among them are long noncoding RNAs (lncRNAs), which are more than 200 nt in length with limited or no protein-coding capacity. LncRNAs are often expressed in a disease-, tissue- or developmental stage-specific manner making these molecules attractive therapeutic targets and pointing toward specific functions for lncRNAs in development and diseases, in particular human cancer [10-13]. Multiple lines of evidence have revealed the contribution of lncRNAs as having oncogenic and tumor suppressor roles in tumorigenesis. A famous oncogenic lncRNA involved in tumor pathogenesis is known as HOTAIR (*Hox transcript antisense intergenic RNA*), which has been consistently upregulated and identified as a strong prognosis marker of patient outcomes such as metastasis and patient survival in diverse human cancers. The studies also revealed that HOTAIR exerts its oncogenic functions via binding the PRC2 (polycomb repressive complex 2), which methylates histone H3 on K27 to promote gene repression [14-16]. A similar mode of action is executed by the lncRNA ANRIL (*antisense non-coding RNA in the INK4 locus*), a novel tumor suppressor interacting with the PRC2 complex to block the activity of *p15*^*INK4B*^, a well-known tumor suppressor gene. Moreover, the depletion of ANRIL increases the expression of *p15*^*INK4B*^ and inhibits cellular proliferation tumorigenesis [17]. Maternally expressed gene 3 (*meg3*) also represents a tumor suppressor gene that encodes a MEG3 lncRNA*,* which expression is lost in an expanding list of primary human tumors, and re-expression of MEG3 could induce cell growth arrest and promote cell apoptosis partly via the activation of P53 [18]. Nevertheless, the overall pathophysiological contributions of lncRNAs to gastric cancer remain largely unknown.

In our current study, which seeks to determine the clinical significance and functions of dysregulated lncRNAs in gastric carcinogenesis, we investigated lncRNA GAS5 (Growth Arrest-Specific Transcript 5), which was previously shown to be consistently downregulated and identified as a tumor-suppressor lncRNA in prostate cancer cells, renal cell carcinoma cells and breast cancer cells [19-21], though its functional significance has not yet been established. In this study, we demonstrated that decreased GAS5 expression was a characteristic molecular change in gastric cancer and investigated the effect of altered GAS5 level on the phenotypes of gastric cancer cells *in vitro* and *in vivo*. Then, we analyzed the potential relationship between this lncRNA level in tumor tissues and existing clinicopathological features of gastric cancer, as well as clinical outcome. Our findings suggest that lncRNA GAS5 may represent a novel indicator of poor prognosis in gastric cancer and may be a potential therapeutic target for diagnosis and gene therapy.

## Methods

### Tissue collection

89 gastric cancer samples were obtained from patients who had underwent surgery at Jiangsu province hospital between 2006 and 2008, and were diagnosed with gastric cancer (stages II, III, and IV; seventh edition of the *AJCC Cancer Staging Manual*) based on histopathological evaluation. Clinical pathology information was available for all samples (Table 1). No local or systemic treatment was conducted in these patients before the operation. All specimens were immediately frozen in liquid nitrogen, and stored at -80°C until RNA extraction. The study was approved by the Research Ethics Committee of Nanjing Medical University, China. Informed consents were obtained from all patients.

**Table 1:** Clinicopathological characteristics and GAS5 expression in 89 patient samples of gastric cancer

**Clinical parameter**	**Number of cases (%)**
**Age (years)**	
<50	46 (51.7)
>50	43 (48.3.)
**Gender**	
Male	53 (59.6)
Female	36 (40.4)
**Location**	
Distal	36 (40.4)
Middle	35 (39.3)
Proximal	18 (20.2)
**Size**	
>5 cm	44 (49.4)
<5 cm	45 (50.6)
**Histologic differentiation**	
Well	6 (6.7)
Moderately	30 (33.7)
Poorly	43 (48.3)
Undifferentiatedly	10 (11.2)
**Invasion depth**	
T1	21 (23.6)
T2	26 (29.2)
T3	23 (25.8)
T4	19 (21.3)
**TNM Stages**	
I	15 (16.9)
II	34 (38.2)
III	35 (39.3)
IV	5 (5.6)
**Lymphatic metastasis**	
Yes	44 (49.4)
No	45 (50.6)
**Regional lymph nodes**	
PN0	45 (50.6)
PN1	16 (18.0)
PN2	18 (20.2)
PN3	10 (11.2)
**Distant metastasis**	
Yes	4 (4.5)
No	85 (95.5)
**Expression of GAS5**	
Low expression	44 (49.4)
High expression	45 (50.6)

### Cell lines and culture conditions

Five gastric cancer cell lines (SGC7901, BGC823, MGC803, MKN45, MKN28), and a normal gastric epithelium cell line (GES-1) were purchased from the Institute of Biochemistry and Cell Biology of the Chinese Academy of Sciences (Shanghai, China). Cells were cultured in RPMI 1640 or DMEM (GIBCO-BRL) medium supplemented with 10% fetal bovine serum (10% FBS), 100 U/ml penicillin, and 100 mg/ml streptomycin in humidified air at 37°C with 5% CO_2_.

### RNA extraction and qRT-PCR analyses

Total RNA was extracted from tissues or cultured cells using TRIzol reagent (Invitrogen, Carlsbad, CA). For qRT-PCR, RNA was reverse transcribed to cDNA by using a Reverse Transcription Kit (Takara, Dalian, China). Real-time PCR analyses were performed with Power SYBR Green (Takara, Dalian China). Results were normalized to the expression of GAPDH. The PCR primers for GAS5 or GAPDH were as follows: GAS5 sense, 5’- CTTCTGGGCTCAAGTGATCCT-3’ and reverse, 5’- TTGTGCCATGAGACTCC ATCAG-3’; GAPDH sense, 5’-GTCAACGGATTTGGTCTGTATT-3’ and reverse, 5’-AGTCTTCTGGGTGGCAGTGAT-3’. qRT-PCR and data collection were performed on ABI 7500. The relative expression of GAS5 was calculated and normalized using the 2^-ΔΔCt^ method relative to GAPDH.

### Plasmid construct

To generate a GAS5 expression vector, the entire sequence of human GAS5 (NR_002578.2, 651 bp) was synthesized and subcloned into pCDNA3.1 vector with incorporate external *NheI* and *BamHI* sites, respectively (Invitrogen, Shanghai, China).

### Transfection of gastric cancer cells

All plasmid vectors (pCDNA3.1-GAS5 and empty vector) for transfection were extracted by DNA Midiprep kit (Qiagen, Hilden, Germany). Gastric cells cultured in six-well plate were transfected with the pCDNA3.1-GAS5, empty vector, si-GAS5 or si-NC using Lipofectamine2000 (Invitrogen, Shanghai, China) according to the manufacturer’s instructions. Cells were harvested after 48 hours for qRT-PCR and western blot analyses. siRNAs for the human GAS5 (1^#^: 5’-CUUGCCUGGACCAGCUUAAUU-3’; 2^#^: CACCAUUUCAACUU CCAG CUUUCUG;3^#^: UACCCAAGCAAGUCAUCCAUGGAUA) and the negative control siRNA (5’-UUCUCCGAACGUGUCACGUUU-3’) were purchased from Invitrogen (Invitrogen, Carlsbad, CA).

### Cell proliferation assays

A cell proliferation assay was performed with MTT kit (Sigma, St. Louis, Mo) according to the manufacturer's instruction. Viable cells were counted by trypan blue staining. For the colony formation assay, cells were placed into 6-well plate and maintained in media containing 10% FBS for 2 weeks. Colonies were fixed with methanol and stained with 0.1% crystal violet (Sigma, St. Louis, Mo). Visible colonies were manually counted.

### Hoechst staining assay

SGC-7901 and BGC-823 cells transfected with pCDNA3.1-GAS5 or empty vector were cultured in six-well cell culture plates, and Hoechst 33342 (Sigma, St Louis, MO, USA) was added to the culture medium; changes in nuclear morphology were detected by fluorescence microscopy using a filter for Hoechst 33342 (365 nm). For quantification of Hoechst 33342 staining, the percentage of Hoechst-positive nuclei per optical field (at least 50 fields) was counted.

### Western blot assay and antibodies

Cells protein lysates were separated by 10% SDS-polyacrylamide gel electrophoresis (SDS-PAGE), transferred to 0.22 μm NC membranes (Sigma) and incubated with specific antibodies. ECL chromogenic substrate was used to visualize the bands and the intensity of the bands was quantified by densitometry (Quantity One software; Bio-Rad, CA, USA). GAPDH antibody was used as control, Anti-E2F1, cyclinD1, P21 and cleaved caspase-3 (1:1000) were purchased from Cell Signaling Technology, Inc (CST).

### Tumor formation assay in a nude mouse model

4 weeks female athymic BALB/c nude mice were maintained under specific pathogen-free conditions and manipulated according to protocols approved by the Committee on the Ethics of Animal Experiments of the Nanjing medical University. SCG7901 cells transfected with pCDNA3.1-GAS5 or empty vector were harvested from six-well cell culture plates, washed with PBS, and resuspended at a concentration of 1 × 10^8^ cells/mL. A volume of 100 μL of suspended cells was subcutaneously injected into a single side of the posterior flank of each mouse. The subcutaneous growth of tumor was examined every three days, and tumor volumes were calculated using the equation V = 0.5 × D × d^2^ (V, volume; D, longitudinal diameter; d, latitudinal diameter) [22]. At 18 days post injection, the mice were sacrificed and tumor weights were measured and also used for further analysis. This study was carried out in strict accordance with the recommendations in the Guide for the Care and Use of Laboratory Animals of the National Institutes of Health.

### Statistical analysis

Statistical analysis was performed using the SPSS software package (version 20.0, SPSS Inc). Statistical significance was tested by a Student’s *t*-test or a Chi-square test as appropriate. Survival analysis was performed using the Kaplan-Meier method, and the log-rank test was used to compare the differences between patient groups.

## Results

### Expression of GAS5 is downregulated in human gastric cancer tissues

**Figure 1:**
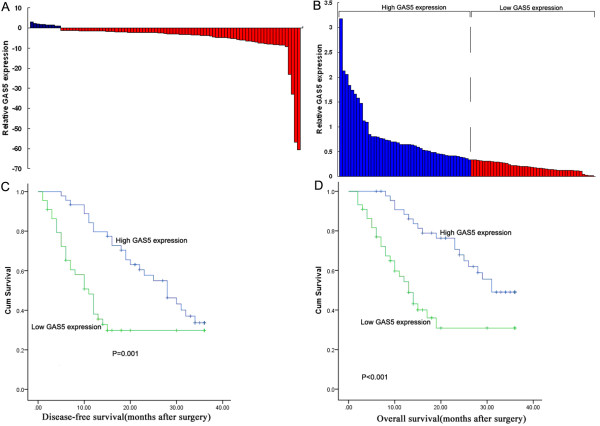
**Relative GAS5 expression in gastric cancer tissues and its clinical significance. (A)** Relative expression of GAS5 in gastric cancer tissues (n = 89) in comparison with corresponding non-tumor normal tissues (n = 89). GAS5 expression was examined by qRT-PCR and normalized to GAPDH expression. Data was presented as fold-change in tumor tissues relative to normal tissues. The red column was defined as overexpression, while the blue as underexpression. **(B)** According to the median ratio of relative GAS5 expression (0.38) in tumor tissues, GAS5 expression was classified into two groups: relative high-GAS5 group (n = 45, blue column) and relative low-GAS5 group (n = 44, red column). **(C, D)** Kaplan-Meier disease-free survival and overall survival curves according to GAS5 expression level.

We firstly examined GAS5 expression level in 89 paired gastric cancer samples and adjacent, histological normal tissues by qRT-PCR, and normalized to GAPDH. Figure 1A showed that the GAS5 level was significantly downregulated in 89% (79/89) gastric cancer tissues compared with corresponding adjacent non-tumorous tissues. In cancerous tissues, GAS5 expression was at a level lower than that of normal specimens, with the median ratio of 0.38 compared with normal counterparts. These data indicate that abnormal GAS5 expression may be related to gastric cancer pathogenesis.

### The relationship between GAS5 expression and clinicopathological factors in patients with gastric cancer

The clinical pathology findings of 89 gastric carcinoma patients are shown in Table 1. According to the median ratio of relative GAS5 expression (0.38) in tumor tissues, the 89 gastric cancer patients were classified into two groups: relative high-GAS5 group (n = 45, GAS5 expression ratio ≥ median ratio) and relative low-GAS5 group (n = 44, GAS5 expression ratio ≤ median ratio) (Figure 1B). Clinicopathologic factors were compared between the two groups (Table 2). The low-GAS5 group was correlated with larger tumor size (p = 0.008), higher TNM stage (*P* < 0.001), deeper depth of invasion (P < 0.001) and more regional lymph nodes (*P* < 0.001) than the high-GAS5 group. However, GAS5 expression level was not associated with other parameters such as gender (*P* = 0.101), age (*P* = 0.338), tumor location (*P* = 0.839), lymphatic metastasis (*P* = 0.072), etc. (Table 2).

**Table 2:** Correlation between GAS5 expression and clinicopathological characteristics in patients with gastric cancer

**Clinical parameter**	**GAS5**		**Chi-squared test** ***P*-value**
	**High- GAS5 group, no. of cases**	**Low-GAS5 group, no. of cases**	
**Age (years)**			
<50	21	25	0.338
>50	24	19	
**Gender**			0.101
Male	23	30	
Female	22	14	
**Location**			0.839
Distal	17	19	
Middle	18	17	
Proximal	10	8	
**Size**			0.008
>5 cm	16	28	
<5 cm	29	16	
**Histologic differentiation**			0.376
Well	3	3	
Moderately	19	11	
Poorly	19	24	
Undifferentiated	4	6	
**Invasion depth**			<0.001
T1	16	5	
T2	18	8	
T3	6	17	
T4	5	14	
**TNM Stage**			0.038
I	11	4	
II	20	14	
III	13	22	
IV	1	4	
**Lymphatic metastasis**			0.072
Yes	27	18	
No	18	26	
**Regional lymph nodes**			<0.001
PN0	28	17	
PN1	12	4	
PN2	3	15	
PN3	2	8	
**Distant metastasis**			0.056
Yes	0	4	
No	45	40	

### Association of GAS5 expression with patients’ survival

We further examined whether GAS5 expression level correlated with outcome of gastric cancer patients after gastrectomy. Disease-free survival (DFS) and overall survival (OS) curves were plotted according to GAS5 expression level by the Kaplan–Meier analysis and log-rank test, respectively, and the results were presented in Figure 1C and D. Remarkably, patients with low GAS5 expression level had poorer disease-free survival (P = 0.001) and overall survival (P < 0.001). With regard to OS, the overall 3-year accumulative survival rates of patients with high GAS5 expression were 49%. For patients with low GAS5 expression, however, the rates were 30.9%. Low GAS5 expression indicated a shorter overall survival time of patients (median OS: 13 months) compared with high GAS5 expression (median OS: 31 months). Moreover, 3 years of disease-free survival for high GAS5 expression was 33.7%, while was 29.8% for low GAS5 expression. The median survival time for high GAS5 expression is 28 months, while is 11 months for low GAS5 expression. These results together suggested downregulated expression of GAS5 in gastric cancer was significantly correlated with patients’ survival time.

### Deregulated expression of GAS5 is an independent prognostic predictor for patient with gastric cancer

In order to estimate the clinical significance of various prognostic factors that might influence survival in the study population, univariate analyses was performed for DFS or OS in 89 patients with gastric cancer, respectively. As shown in Table 3, TNM stage, distant metastasis and GAS5 expression were statistically significant risk factors affecting DFS or OS of patients. The other clinicopathological features, such as age, gender, tumor location, and tumor size were not statistically significant prognosis factors (*P* > 0.05). Low intratumoral GAS5 expression is a significant negative predictor for DFS (hazard ratio [HR], 0.407; 95% confidence interval [CI], 0.235 to 0.706; *P* = 0.001) or OS (HR, 0.591; 95% CI, 0.436 to 0.803; *P* = 0.001). To evaluate the robustness of the prognostic value of intratumoral GAS5 expression, variables with a value of *P* < 0.05 were selected for multivariate analysis. As shown in Table 3, multivariate analysis revealed that GAS5 expression and TNM stage were independent prognostic markers for gastric cancer. Taken together, these data indicate that low GAS5 expression level is an independent risk factor for gastric cancer patients.

**Table 3:** Univariate and multivariate Cox regression analyses GAS5 for DFS or OS of patients in study cohort (n = 89)

**Variables**	**DFS**			**OS**		
	**HR**	**95% CI**	***P*** **value**	**HR**	**95% CI**	***P*** **value**
**Univariate analysis**					.	
Age (<50 years vs. >50 years)	1.122	0.659-1.910	0.671	1.057	0.589-1.899	0.852
Gender (male vs. female)	1.584	0.907-2.768	0.106	1.739	0.933-3.240	0.082
Location (Distal vs. Middle + Proximal)	1.232	0.811-1.872	0.327	1.498	0.830-2.702	0.180
tumor size (>5 cm vs. <5 cm)	1.493	0.876-2.543	0.141	1.260	0.938-1.692	0.124
Histologic differentiation (Well + Moderately vs. Poorly + Undifferentiated)	0.743	0.428-1.288	0.289	0.798	0.436-1.458	0.463
Invasion depth (T1 + T2 vs.T3 + T4)	0.629	0.370-1.069	0.087	0.724	0.403-1.300	0.279
TNM stage (I + II vs. III + IV)	0.521	0.303-0.896	0.018**	0.433	0.239-0.786	0.006**
Lymphatic metastasis (No vs. Yes)	0.838	0.642-1.093	0.192	0.899	0.671-1.204	0.474
Regional lymph nodes (PN0+ PN1vs. PN2+ PN3)	0.584	0.332-1.024	0.061	0.575	0.310-1.065	0.078
Distant metastasis (No vs. Yes)	0.456	0.264-0.787	0.005**	0.432	0.231-0.811	0.009**
Expression of GAS5 (High vs. Low)	0.407	0.235-0.706	0.001**	0.591	0.436-0.803	0.001**
**Multivariate analysis**						
TNM stage (I + II vs. III + IV)	0.631	0.357-1.115	0.113	0.537	0.289-1.000	0.049*
Distant metastasis (No vs. Yes)	0.384	0.123-1.197	0.099	0.381	0.105-1.389	0.144
Expression of GAS5 (High vs. Low)	0.466	0.263-0.828	0.009**	0.412	0.218-0.776	0.006**

DFS, disease-free survival; OS, overall survival; HR, hazard ratio; 95% CI, 95% confidence interval. *P < 0.05 and **P < 0.01.

### Manipulation of GAS5 expression level in gastric cancer cells

**Figure 2:**
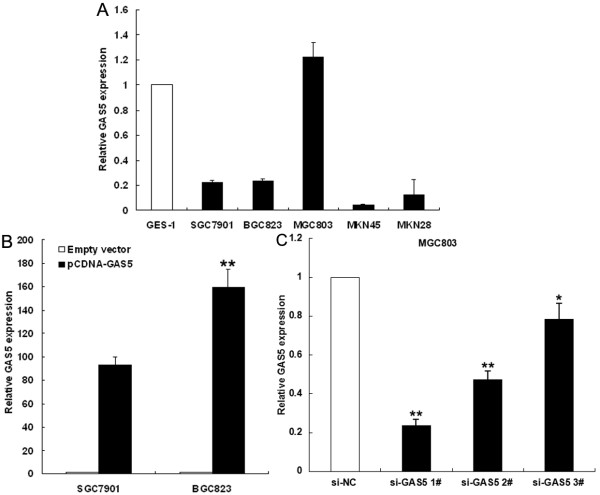
**The level of GAS5 expression in gastric cancer cells. (A)** Results from qRT-PCR demonstrating GAS5 expression level of gastric cancer cell lines (SGC7901, BGC823, MGC803, MKN28 and MKN45) compared with normal human gastric epithelial cell line (GES-1). **(B)** qRT-PCR analyses of GAS5 expression level following treatment BGC823 and SGC7901 cells with pCDNA3.1-GAS5 or empty vector. **(C)** qRT-PCR analyses of GAS5 expression level following treatment MGC803 cells with si-GAS5 or si-NC. **P < 0.01.

To evaluate the biological functions of GAS5, we first examined the expression of GAS5 in a variety of cell lines, including SGC7901, BGC823, MGC803, MKN45, MKN28, and normal gastric epithelium cell line GES-1, by qRT-PCR. The results showed that GAS5 expression was obviously downregulated in gastric cancer cells (Figure 2A), suggesting that a decrease in expression levels may be significant in gastric carcinogenesis.

In order to manipulate GAS5 level in gastric cancer cells, pCDNA3.1-GAS5 vector was transfected into BGC823 and SGC7901 cells. Expression of GAS5 was assessed using qRT-PCR analysis and a respective 159-fold and 93-fold increase in the pCDNA3.1-GAS5-transfected cells compared with the vector controls (Figure 2B). Furthermore, GAS5 siRNAs was transfected into MGC803 cells to downregulate endogenous GAS5 expression, qRT-PCR analysis revealed that GAS5 expression was effectively knocked down in si-GAS5 transfected cells when compared with si-NC control cells (Figure 2C).

### Effect of GAS5 on gastric cancer cell proliferation and apoptosis *in vitro*

**Figure 3:**
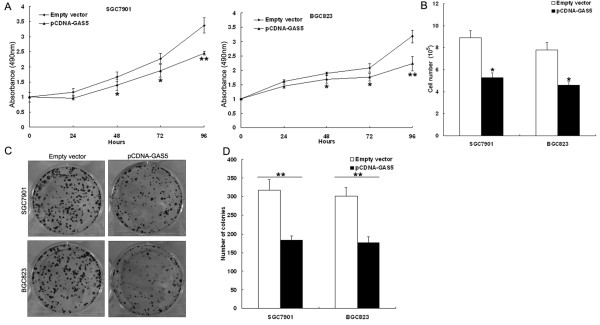
**Ectopic expression of GAS5 inhibits gastric cancer cell proliferation *in vitro*.** SGC7901 and BGC823 cells were transfected with pCDNA3.1-GAS5 vector (or empty vector), respectively. **(A)** MTT assay was performed to determine the proliferation of pCDNA3.1-GAS5 transfected SGC7901 and BGC823 cells. Data represent the mean ± s.d. from three independent experiments. **(B)** Viable cells were counted by trypan blue staining at 72 h after transfection. **(C, D)** Colony-forming growth assay was performed to determine the proliferation of pCDNA3.1-GAS5 transfected SGC7901 and BGC823 cells. The colonies were counted and captured. **P < 0.01.

**Figure 4:**
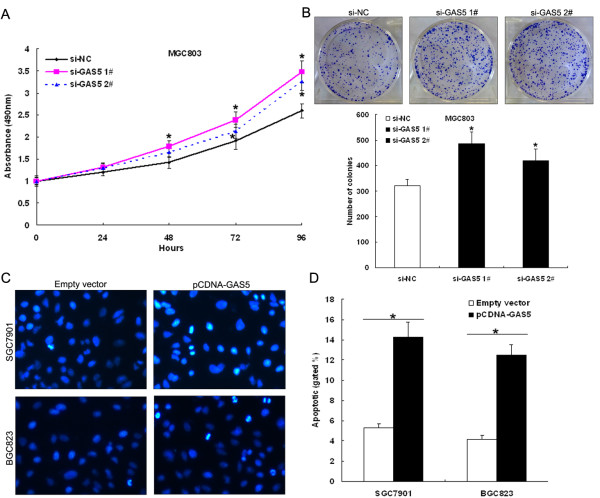
**Downregulation of endogenous GAS5 promotes gastric cancer cell proliferation. (A)** MTT assay was performed to determine the proliferation of si-GAS5 transfected MGC803 cells. **(B)** Colony-forming growth assay was performed to determine the proliferation of si-GAS5 transfected MGC803 cells. **(C, D)** The effect of GAS5 on gastric cancer cells apoptosis. SGC7901 and BGC823 cells were transfected with pCDNA3.1-GAS5 vector (or empty vector), respectively. Hoechst staining assay for cell apoptosis; the percentage of Hoechst-positive nuclei per optical field (at least 50 fields) was counted. *P < 0.05.

To assess the biological role of GAS5 in gastric cancer, we investigated the effect of targeted knockdown or overexpression of GAS5 on cell proliferation and apoptosis. MTT assay and trypan blue staining revealed that cell growth was significantly impaired in pCDNA3.1-GAS5 transfected SGC7901 cells or BGC823 cells (Figure 3A and B), while proliferation of MGC803 cells was increased in si-GAS5 transfected cells compared with respective controls (Figure 4A). Similarly, the results of colony-formation assays revealed that clonogenic survival was decreased following upregulation of GAS5 in pCDNA3.1-GAS5 transfected SGC7901 cells or BGC823 cells (Figure 3C and D), while enhanced in si-GAS5 transfected MGC803 cells (Figure 4B).

**Figure 5:**
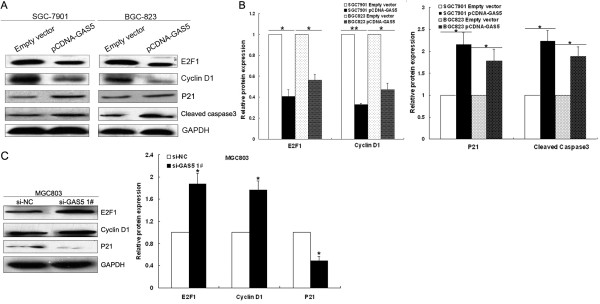
**GAS5 affects E2F1 and P21 protein levels. (A, B)** Western blot analysis of E2F1, CyclinD1, P21 and cleaved caspase-3 in pCDNA3.1-GAS5 transfected SGC7901 or BGC823 cells and respective control cells. **(C)** Western blot analysis of E2F1, CyclinD1 and P21 after si-GAS5 transfection with MGC 803 cells. GAPDH protein was used as an internal control. * *P* < 0.05; ** *P* < 0.01.

To determine whether apoptosis was a contributing factor to cell growth inhibition, we performed Hochest staining analysis of pCDNA3.1-GAS5 transfected SGC7901 and BGC823 cells. The results showed that the number of cells with condensed and fragmented nuclei indicating the fraction of early apoptotic cells was significantly different in SGC7901 and BGC823 cells with pCDNA3.1-GAS5 transfection compared with empty vector transfected cells (Figure 4C and D). In addition, we found that forced expression of GAS5 enhanced caspase-3-dependent apoptosis, demonstrated by western blot analysis of activated caspase-3 after pCDNA3.1-GAS5 transfection (Figure 5A and B). Taken together, these results indicate that upregulation of GAS5 suppresses gastric cancer cell proliferation, and induces cell apoptosis *in vitro*.

### GAS5 inhibits gastric cancer cells tumorigenesis *in vivo*

**Figure 6:**
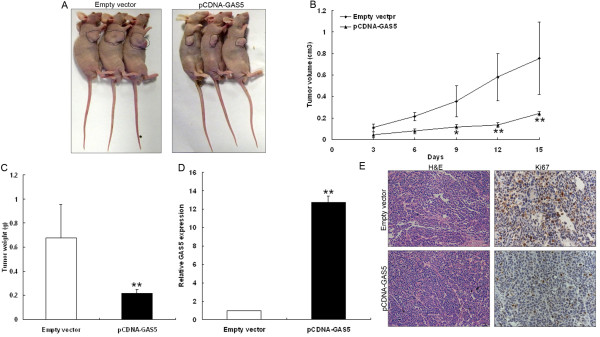
**Effects of GAS5 on tumor growth *in vivo*. (A, B)** The tumor volume was calculated every three days after injection of SGC7901 cells stably transfected with pCDNA3.1-GAS5 or empty vector. Points, mean (n = 3); bars indicate S.D. **(C)** Tumor weights were represented as means of tumor weights ± s.d. **(D)** qRT-PCR analysis of GAS5 expression in tumor tissues formed from SGC7901/pCDNA3.1-GAS5, SGC7901/Empty vector. **(E)**. Tumors developed from pCDNA3.1-GAS5 transfected SGC7901 cells showed lower Ki67 protein levels than tumors developed by control cells. Upper: H & E staining; Lower: immunostaining. * *P* < 0.05; ** *P* < 0.01.

To explore whether the level of GAS5 expression could affect tumorigenesis, pCDNA3.1-GAS5 or empty vector stably-transfected SGC7901 cells were inoculated into female nude mice. Eighteen days after injection, the tumors formed in pCDNA3.1-GAS5 group were substantially smaller than those in the control group (Figure 6A and B). Moreover, the mean tumor weight at the end of the experiment was markedly lower in the pCDNA3.1-GAS5 group (0.21 ± 0.03 g) compared to the empty vector group (0.67 ± 0.28 g) (Figure 6C). qRT-PCR analysis of GAS5 expression was then performed in selected tumor tissues. The results showed that the levels of GAS5 expression in tumor tissues formed from pCDNA3.1-GAS5 cells were higher than those of tumors formed in control group (Figure 6D). Immunostaining was used to analyze Ki67 protein expression in resected tumor tissues. Ki67 levels in tumors formed from pCDNA3.1-GAS5 transfected SGC7901 cells, exhibited decreased positivity for Ki67 than in tumors from control cells (Figure 6E). These results indicate that overexpression of GAS5 could inhibit tumor growth *in vivo*.

### E2F1 and P21 are key downstream mediators of GAS5

Further exploration of the underlying mechanisms involved in GAS5 overexpression induced growth arrest was done by examining the expression of potential targets after transfection with pCDNA3.1-GAS5 or empty vector. The results showed that the expression of E2F1 was significantly decreased and the expression of cyclin D1 was also downregulated in gastric cancer cells transfected with pCDNA3.1-GAS5 compared to those with empty vector. Moreover, increased P21 protein level were observed in cells transfected with pCDNA3.1-GAS5 compared to those with empty vector (Figure 5A and B). However, the mRNA expression of E2F1, cyclinD1 or P21 remained unaltered in the GAS5-overexpressed gastric cancer cells compared with the vector controls (data not shown). Meanwhile, we also assayed for changes in the protein expression of E2F1, cyclinD1 and P21 in si-GAS5 transfected MGC803 cells. As expected, when compared with si-NC control cells, inhibition of GAS5 resulted in an increase in E2F1 and cyclinD1 and a decrease of P21 levels (Figure 5C). These data suggest that GAS5 maybe function as an tumor suppressor by regulating E2F1 and P21 through post-transcriptional regulation, and further experiments are needed to elucidate the potential mechanism.

## Discussion

LncRNAs dysregulation may affect epigenetic information and provide a cellular growth advantage, resulting in progressive and uncontrolled tumor growth [14-18]. Effective control of both cell survival and cell proliferation is critical to the prevention of oncogenesis and to successful cancer therapy. Therefore, identification of cancer-associated lncRNAs and investigation of their clinical significance and functions may provide a missing piece of the well-known oncogenic and tumor suppressor network puzzle.

GAS5 is a long ncRNA (~650 bases in humans) that was originally isolated from a screen for potential tumor suppressor genes expressed at high levels during growth arrest [23]. Its encoding gene, *gas5*, comprises 12 exons and encodes ten box C/D snoRNAs within its introns [24]. Two mature GAS5 lncRNAs, GAS5a and GAS5b, have also been identified in humans due to the presence of alternative 5’-splice donor sites in exon 7, whereas GAS5b is the major isoform (NR_002578.2, 77 nt, simply called GAS5 in this study), and GAS5a has only 45 nt, missing 32 nt at the 3’ end [25]. GAS5 has been shown to be aberrantly expressed in prostate cancer, renal cell carcinoma, breast cancer, head and neck squamous cell carcinoma (HNSCC), and glioblastoma multiforme [19-21,26]. For breast cancer and HNSCC, low GAS5 expression is an adverse prognostic factor for survival. Moreover, overexpression of GAS5 attributed to growth arrest of several cancer cell lines through regulation of apoptosis and cell cycle, under basal conditions or various cell death stimuli, including chemotherapeutic agents, suggesting its clinical significance in the development and therapy of cancer [19-21]. These data demonstrate the potential tumor-suppressor role of GAS5; however, the relationship between expression of GAS5 and gastric cancer development and/or progression remains unclear.

Our studies were designed to investigate the expression and prognostic significance of GAS5 in patients with gastric cancer. GAS5 expression was retrospectively analyzed in 89 patients with gastric carcinoma. Results were assessed for association with clinical features and DFS/OS of gastric cancer patients after gastrectomy. Prognostic values of GAS5 expression and clinical outcomes were also evaluated by Cox regression analysis. The results showed that GAS5 expression was significantly decreased in gastric cancer tissues and cell lines. A lower expression of GAS5 was detected in tumor of larger size, higher tumor stage, deeper depth of invasion and more regional lymph nodes. In addition, the downregulation expression of GAS5 was associated with poor prognosis. Moreover, ectopic expression of GAS5 was demonstrated to decrease gastric cancer cell proliferation and induce apoptosis, while downregulation of endogenous GAS5 could promote cell proliferation *in vitro* and *in vivo*. Taken together, these findings indicate that GAS5 could function as a tumor suppressor via regulating cell growth and apoptosis, and may be useful in the development of novel prognostic or progression markers for gastric cancer.

Although GAS5 has been suggested to have a tumor-suppressive role, the underlying mechanism of GAS5-mediated gene expression having an impact on tumorigenesis is still elusive. Kino *et al.* have found that GAS5 could structurally mimic the glucocorticoid receptor response element (GRE) to suppress GR-induced transcriptional activity of endogenous glucocorticoid- responsive genes [25]. Zhang *et al.* have provided a possible mechanism for GAS5 as a tumor suppressor, which may be attributed to its ability to suppress the oncogenic miR-21 in breast cancer [27]. Nevertheless, since it’s highly possible that target genes of lncRNAs differ between specific tissues and cell types, specific target genes controlled by GAS5 for gastric pathogenesis remain unknown and deserve investigation. In this study, to explore the molecular mechanism by which GAS5 contributes to cell proliferation of gastric cancer, we investigated potential targets which were responsible for cell cycle arrest and cell growth inhibition. Our present experimental results confirmed that E2F1, as well as Cyclin D1, were functional targets of GAS5 in gastric cells. E2F1 expression has been found to be upregulated in mutiple cancers, and its overexpression contributes to many tumors development by acting as an important transcript factor regulating key regulator genes that controlling cell proliferation [28,29]. Cyclin D1 is one of the most important proteins to regulate cell cycle, and related with the development of many cancers. Cyclin D1 binds and activates CDK4/6, which subsequently phosphorylates tumor suppressor protein Rb and allows the cell cycle to progress through G1 into S [30]. Furthermore, P21 expression has been shown to be reduced or lost in a variety of cancer types [31]. A possible explanation is that P21 exerts its inhibitory control over the cell cycle primarily through direct binding to cyclins and CDKs, therefore preventing cell proliferation [32]. Here, we also found P21 was a downstream regulator involved in GAS5-mediated growth arrest in gastric cancer cells. Taken together, these findings indicate that lncRNA GAS5 may function as a tumor suppressor and its deficiency or decreased expression could contribute to gastric cancer development; however, further studies are required to clarify GAS5 regulation of the above targets expression in gastric cancer cells.

## Conclusion

In summary, we demonstrate that the decreased GAS5 expression is a common event underlying gastric cancer, indicating that GAS5 may play a key tumor-suppressive as an indicator of poor survival rate and a negative prognostic factor for gastric cancer patients. Further well understanding of the mechanisms of GAS5 in the molecular etiology of gastric cancer will promote the development of lncRNA-directed diagnostic and therapeutic agents against this deadly disease.

## Competing interests

The authors declare that they have no competing interests.

## Authors’ contributions

SM, KR, DW and LXH were involved in the conception and design of the study. JFY, ZEBand LJH were involved in the provision of study material and patients. LYW, XR and XTP performed the data analysis and interpretation. SM wrote the manuscript. LXH and DW approved the final version. All authors read and approved the final manuscript.

## Authors’ information

Ming Sun, Fei-yan Jin, and Rui Xia are joint first authors.

## Pre-publication history

The pre-publication history for this paper can be accessed here:

http://www.biomedcentral.com/1471-2407/14/319/prepub
